# A Thyroid Abscess Developed Three Months After a Fine Needle Aspiration of a Colloid Cyst: A Case Report

**DOI:** 10.7759/cureus.108349

**Published:** 2026-05-06

**Authors:** Andrijana Jovic, Ines Strbacko, Jerko Bilos

**Affiliations:** 1 Department of Diagnostic and Interventional Neuroradiology, University Hospital Centre Zagreb, Zagreb, HRV; 2 Department of Otorhinolaryngology, Head and Neck Surgery, University Hospital Centre Zagreb, Zagreb, HRV

**Keywords:** acute suppurative thyroiditis, colloid cyst, ct, head and neck pathologies, thyroid abscess

## Abstract

Fine needle aspiration (FNA) is widely used as a first-line diagnostic procedure for thyroid nodules and cysts and is considered safe, with serious complications reported rarely. Thyroid infections and abscesses are uncommon because of the gland’s anatomical isolation, rich vascular supply, lymphatic drainage, and intrinsic antimicrobial properties. We report a delayed thyroid abscess that developed three months after fine-needle aspiration of a colloid cyst in an immunocompetent patient. Such a delayed presentation is unusual and highlights the importance of considering late infectious complications following this routine procedure.

A 39-year-old woman presented with a five- to six-day history of anterior neck pain, redness, and fever up to 38.8°C. She had undergone ultrasound-guided fine needle aspiration of a colloid thyroid cyst three months prior. Clinical examination revealed an erythematous, edematous, and indurated swelling over the thyroid region. Laboratory findings showed leukocytosis and markedly elevated inflammatory markers, while thyroid function remained normal. Imaging demonstrated a large cystic lesion in the left thyroid lobe with rim enhancement and tracheal compression.

The patient underwent urgent surgical management with drainage of purulent material and left hemithyroidectomy, followed by intravenous antibiotic therapy. Histopathology confirmed a thyroid abscess. The postoperative course was uneventful, and the patient was discharged in good condition. Thyroid abscess may occur as a rare and delayed complication after fine needle aspiration, even in immunocompetent patients. Progressive anterior neck swelling and systemic inflammatory response following aspiration warrant prompt imaging and early surgical and antibiotic management to prevent airway compromise and other life-threatening complications.

## Introduction

FNA is generally considered safe and carries a low risk of serious complications. It is widely used as a first-line diagnostic method for thyroid nodules and cysts [1,2]. The technique involves inserting a thin needle, typically 22-27 gauge, into the lesion to obtain cellular material for cytological analysis. The procedure is often performed under ultrasound (US) guidance to improve accuracy and ensure precise needle placement. The most common complications include mild pain, discomfort, and a minor hematoma at the aspiration site. More serious and potentially life-threatening complications, such as infection, thyroid hematoma, intracapsular hemorrhage, and acute thyroid swelling, have been described but are considered very rare [3-5].

While most reported cases describe an acute onset of thyroid abscess shortly after FNA, delayed presentations have also been documented [6]. Early recognition of these rare complications is crucial, as they may significantly worsen the clinical course and, in severe cases, lead to fatal outcomes [7]. In this case report, we present a patient who developed a thyroid abscess as a rare complication following fine needle aspiration of a colloid cyst.

## Case presentation

A 39-year-old woman was admitted to our emergency department with redness and discomfort in the anterior neck region and a fever of up to 38.8°C lasting five to six days. Three months earlier, a fine-needle aspiration of a colloid cyst within a goiter had been performed and verified by ultrasound and cytology. Earlier that day, laboratory tests showed normal thyroid function parameters and markedly elevated C-reactive protein (CRP) levels (420 mg/L). She denied difficulty breathing or swallowing, chest pressure, nausea, or vomiting. Her past medical history was unremarkable, with no chronic medication use and no known drug allergies. She is a smoker and consumes alcohol occasionally.

On inspection, the anterior neck was edematous and erythematous with visible swelling in the thyroid region. On palpation, the area was hyperthermic and indurated but painless. Neurocirculatory status was normal. On physical examination, the patient was afebrile, with elevated blood pressure (178/118 mmHg), tachycardia (149 beats per minute (bpm)), oxygen saturation of 97% on room air, a respiratory rate of 16 breaths per minute, and a normal ECG. Otorhinolaryngological examination revealed a normal appearance of the mucosa of the oral cavity and pharynx. Fiberendoscopy demonstrated normally mobile vocal cords with an adequate rima glottidis, along with normal laryngeal and hypopharyngeal mucosa. The remainder of the physical examination, including the chest, abdomen, extremities, and neurological assessment, was unremarkable. Laboratory evaluation revealed leukocytosis with an elevated white blood cell count (15.6 × 10⁹/L), neutrophils at 77.2%, and an elevated C-reactive protein (CRP) level (406.0 mg/L). Laboratory findings at presentation are summarized in Table 1.

**Table 1:** Laboratory findings at presentation

Parameter	Patient Value	Reference Range
White blood cell count (WBC)	15.6 × 10⁹/L	4.0–10.0 × 10⁹/L
Neutrophils	77.2%	40–70%
C-reactive protein (CRP)	406–420 mg/L	<5 mg/L
Thyroid-stimulating hormone (TSH)	Within normal limits	0.4–4.0 mIU/L
Free thyroxine (fT4)	Within normal limits	9–19 pmol/L

**Figure 1:**
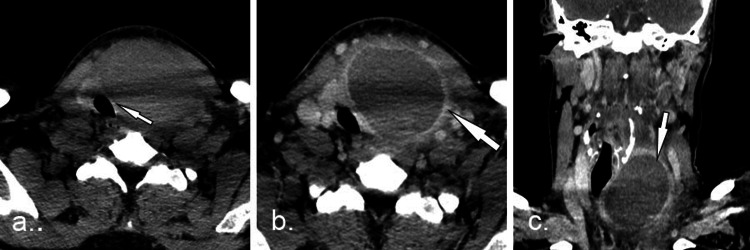
CT images of the patients neck (a) Axial non-contrast images showing a mass in the left infrahyoid neck displacing the trachea to the right (white arrow). (b) Axial contrast-enhanced images demonstrating ring enhancement of the lesion (arrow) with surrounding edema. (c) Coronal images depicting the abscess (arrow).

An emergency contrast-enhanced neck CT subsequently demonstrated a peripheral (ring) enhancing cystic lesion within the left thyroid lobe measuring 60 mm in diameter and containing dense material, with surrounding edema extending into the left retropharyngeal space. The remaining thyroid parenchyma had normal morphology. The visceral structures of the neck and trachea were displaced to the right and compressed, with the narrowest tracheal diameter measuring 9 mm. Bilateral lymph nodes in regions III-IV were enlarged but morphologically regular. The findings were consistent with an abscess in the left thyroid lobe (Figure 1).

After confirmation of the diagnosis, urgent surgery was performed on the same day. To reduce tension, aspiration of purulent material was performed first, followed by left hemithyroidectomy and exploration of the retropharyngeal and parapharyngeal spaces, which revealed no additional abscess collections. A suction drain was placed intraoperatively. Intravenous antibiotic therapy was initiated with clindamycin (3 × 600 mg) and cefuroxime (3 × 1.5 g), along with appropriate analgesia and supportive therapy for a total duration of four days. Histopathological examination of the left thyroid lobe confirmed the diagnosis of a thyroid abscess with acute suppurative inflammation. On postoperative day 4, the patient was discharged home in good general condition with a recommendation for continued oral clindamycin MIP (2 × 600 mg) for an additional six days, along with a probiotic.

## Discussion

Infections of the thyroid, particularly abscesses, are very rare in both children and adults, primarily due to the anatomical and physiological characteristics of the gland itself [8,9]. A fibrous capsule and fascial planes separate the gland from surrounding structures and prevent local spread of pathogens, while its rich blood supply and lymphatic drainage, as well as high iodine and hydrogen peroxide content, inhibit microbial growth and provide resistance to infection [10,11]. Therefore, it is not surprising that thyroid abscesses and acute suppurative thyroiditis account for less than 0.7% of surgically treated thyroid pathologies [12].

A primary thyroid abscess most commonly occurs as a sequela of acute suppurative thyroiditis (AST), often in patients with congenital abnormalities such as a pyriform sinus fistula or thyroglossal duct cyst [13,14], although the route or source of infection is frequently not identified [15]. Predisposing factors also include underlying thyroid disease, such as Hashimoto’s thyroiditis or thyroid malignancy, sepsis, and trauma, with Staphylococcus and Streptococcus species most commonly reported as causative agents. Immunocompromised patients, those receiving immunosuppressive therapy, and individuals with HIV infection, diabetes, or inherited immunodeficiency are particularly predisposed to this condition [16,17].

Our patient was immunocompetent and, apart from the thyroid disorder, had no significant past medical history and was not receiving chronic therapy. She underwent a fine needle aspiration procedure for a colloid cyst three months earlier. The development of a thyroid abscess following fine-needle aspiration has been described in immunodeficient patients; however, in an immunocompetent host, particularly with a three-month delay, it is considered rare and unusual [18,19]. While most reported cases describe an acute onset of thyroid abscess shortly after FNA, delayed and chronic presentations have also been reported. Purdy et al. reported a case of thyroid abscess occurring four weeks after FNA biopsy, supporting the concept that infectious complications may present in a delayed, subacute manner rather than acutely [6].

Syed et al. described a case of chronic suppurative thyroid abscess developing approximately one year after FNAC, characterized by persistent swelling and a discharging sinus tract. This supports the possibility of a prolonged and indolent infectious process. In comparison, our case demonstrated abscess formation three months after FNA, representing an intermediate but still delayed presentation [20].

Our patient presented with redness and discomfort in the anterior neck region accompanied by fever, which are common features of thyroid infections and abscesses, although she did not develop other typical signs such as localized pain or cervical lymphadenopathy [20]. Other commonly reported symptoms include dysphagia, odynophagia, dyspnea, ear pain, and hoarseness. In laboratory evaluation, as in our case, leukocytosis and elevated CRP levels are typical findings, while thyroid function usually remains normal.

When thyroid pathology is suspected, ultrasound and CT are commonly used imaging modalities in the diagnostic process [9]. Due to markedly elevated inflammatory parameters and strong clinical suspicion of thyroiditis, our patient underwent urgent contrast-enhanced CT of the neck to evaluate for infection or abscess, which was confirmed radiologically. She had no congenital anomalies that would predispose her to abscess formation and did not develop severe complications, although inflammation and edema resulted in rightward displacement and compression of the trachea, with a minimal diameter of 9 mm.

The differential diagnosis of anterior neck swelling is broad and includes thyroid conditions such as acute, subacute, or chronic thyroiditis and abscess, aggressive thyroid malignancy, cyst rupture or nodule hemorrhage; parathyroid hemorrhage or abscess; neck trauma; sternocleidomastoid abscess; cervical lymphadenitis; branchial cleft cyst; and inflamed thyroglossal duct cyst, among others [13]. Given the broad differential diagnosis and complex anatomical relationships in the neck, CT is often preferred as the initial imaging modality in acutely ill patients with suspected acute suppurative thyroiditis, as it delineates the extent and complexity of abscess formation.

Complications of thyroid abscess are serious and potentially fatal, including abscess rupture, extension to the pharynx, neck, and mediastinum [7,9,10], airway compromise, thyrotoxicosis, internal jugular vein thrombosis, and sepsis. Therefore, early diagnosis and prompt treatment are essential. Furthermore, histopathological evaluation of abscess material is important, as abscess formation may occasionally be associated with underlying thyroid malignancy. Treatment of a thyroid abscess must be individualized but typically includes antibiotic therapy and surgical intervention, such as drainage, hemithyroidectomy in selected cases, or total thyroidectomy in severe cases [7]. Due to the large abscess size (60 mm) and extensive inflammation, our patient underwent hemithyroidectomy combined with antibiotic therapy and was discharged four days later in good general condition.

## Conclusions

Thyroid abscess is a rare but potentially life-threatening condition that may occur as a delayed complication following fine needle aspiration, even in immunocompetent patients without predisposing factors. Although fine needle aspiration is widely regarded as a safe and routine diagnostic procedure, clinicians should remain vigilant for late-onset infectious complications, particularly in patients presenting with progressive anterior neck swelling and systemic inflammatory signs. Prompt recognition, appropriate imaging, and early surgical and antibiotic management are essential to prevent airway compromise and other serious complications. Awareness of this rare presentation may facilitate timely diagnosis and improve clinical outcomes.
